# T-cell-mediated regulation of neuroinflammation involved in neurodegenerative diseases

**DOI:** 10.1186/s12974-014-0201-8

**Published:** 2014-12-02

**Authors:** Hugo González, Rodrigo Pacheco

**Affiliations:** Laboratory of Neuroimmunology, Fundación Ciencia & Vida, Avenida Zañartu #1482, Ñuñoa 7780272 Santiago, Chile; Departamento de Ciencias Biológicas, Facultad de Ciencias Biológicas, Universidad Andrés Bello, 8370146 Santiago, Chile

**Keywords:** Neuroinflammation, Neurodegenerative disorders, Microglia, CD4+ T-cells

## Abstract

Neuroinflammation is involved in several neurodegenerative disorders and emerging evidence indicates that it constitutes a critical process that is required for the progression of neurodegeneration. Microglial activation constitutes a central event in neuroinflammation. Furthermore, microglia can not only be activated with an inflammatory and neurotoxic phenotype (M1-like phenotype), but they also can acquire a neurosupportive functional phenotype (M2-like phenotype) characterised by the production of anti-inflammatory mediators and neurotrophic factors. Importantly, during the past decade, several studies have shown that CD4^+^ T-cells infiltrate the central nervous system (CNS) in many neurodegenerative disorders, in which their participation has a critical influence on the outcome of microglial activation and consequent neurodegeneration. In this review, we focus on the analysis of the interplay of the different sub-populations of CD4^+^ T-cells infiltrating the CNS and how they participate in regulating the outcome of neuroinflammation and neurodegeneration in the context of Parkinson’s disease, Alzheimer’s disease, amyotrophic lateral sclerosis and multiple sclerosis. In this regard, encephalitogenic inflammatory CD4^+^ T-cells, such as Th1, Th17, GM-CSF-producer CD4^+^ T-cells and γδT-cells, strongly contribute to chronic neuroinflammation, thus perpetuating neurodegenerative processes. In contrast, encephalitogenic or meningeal Tregs and Th2 cells decrease inflammatory functions in microglial cells and promote a neurosupportive microenvironment. Moreover, whereas some neurodegenerative disorders such as multiple sclerosis, Parkinson’s disease and Alzheimer’s disease involve the participation of inflammatory CD4^+^ T-cells 'naturally', the physiopathology of other neurodegenerative diseases, such as amyotrophic lateral sclerosis, is associated with the participation of anti-inflammatory CD4^+^ T-cells that delay the neurodegenerative process. Thus, current evidence supports the hypothesis that the involvement of CD4^+^ T-cells against CNS antigens constitutes a key component in regulating the progression of the neurodegenerative process.

## Introduction

The neuroinflammatory process has been associated with most neurodegenerative diseases including Alzheimer’s disease (AD), Parkinson’s disease (PD), multiple sclerosis (MS) and amyotrophic lateral sclerosis (ALS) [1]. Furthermore, emerging evidence indicates that neuroinflammation constitutes a critical process for the progression of neurodegeneration involved in neurodegenerative disorders [2]. Microglial activation plays a central role in neuroinflammation, with microglial cells being the main source of reactive oxygen species (ROS) and nitrogen species, glutamate and TNF-α, all of which are highly neurotoxic when released in high doses by activated microglia [2–5].

Several studies have shown that microglial activation may be evoked by the stimulation of toll-like receptors (TLRs) through the aggregated proteins in the central nervous system (CNS) of individuals with neurodegenerative diseases, as well as in animal models [6–9]. For instance, a pathological hallmark in the brain of AD patients includes extracellular deposition of the fibrillar form of β-amyloid peptide (Aβ) surrounded by dystrophic neurites, forming senile plaques and intracellular neurofibrillary tangles constituted of hyperphosphorylated forms of the microtubule-binding protein Tau [10]. Similarly to AD, PD is a proteinopathy that is characterised by the accumulation and aggregation of misfolded α-synuclein and ubiquitin in cytoplasmic inclusions called Lewy bodies and Lewy neurites. These cytoplasmic inclusions are found in both sporadic and inherited forms of PD [11]. Similarly, ALS involves the aggregation of superoxide dismutase 1 (SOD1) in the CNS [12]. Other examples include associations of aggregated huntingtin in Huntington’s disease, Prp-amyloid in prion disease and Reelin in aging brain [1].

Similar to the functional behaviour of peripheral macrophages [2], microglia can not only be activated with an inflammatory and neurotoxic phenotype (M1-like phenotype), but they can also acquire a neurosupportive functional phenotype (M2-like phenotype), characterised by the production of anti-inflammatory mediators and neurotrophic factors, including insulin-like growth factor 1 (IGF-1), brain-derived neurotrophic factor (BDNF), glial cell-derived neurotrophic factor (GDNF), among others [13–15]. Intercellular interactions between microglia and other cellular factors play a fundamental role in the outcome of the functional phenotype acquired by activated microglia and, therefore, in neurodegeneration. Cellular factors influencing microglial fate include astrocytes, neurons, epithelial cells of the blood-brain barrier (BBB) and T-cells infiltrating the CNS [2]. Indeed, during the last decade, several studies have shown that CD4^+^ T-cells infiltrate the CNS in many neurodegenerative disorders, and that their participation has a critical influence on the outcome of microglial activation and consequent neuronal damage [1,2].

The precise role of CD4^+^ T-cells infiltrating the CNS in the outcome of neuroinflammation strongly depends on the functional phenotype of these cells [2]. Naïve CD4^+^ T-cells may be activated by antigen-presenting cells (APCs) in the presence of diverse mediators and, depending on the precise composition of the mediators *milieu*, they can differentiate different functional phenotypes, each of them specialised in orchestrating an immune responses against a different kind of threat. For instance, in the presence of IL-12, the differentiation of CD4^+^ T-cells toward the T-helper 1 (Th1) phenotype is favoured, representing a functional phenotype specialised in the elimination of intracellular pathogens. Furthermore, this inflammatory phenotype has been associated with neuroinflammation and neuronal damage [16,17]. The acquisition of the inflammatory Th1 phenotype is controlled by the master transcription factor Tbet, and the main effector cytokine produced by these cells is IFN-γ [18]. Another important inflammatory functional phenotype associated with neuroinflammation and neurodegeneration is the Th17 phenotype, which is favoured by the presence of IL-23 during the activation of naïve CD4^+^ T-cells. These cells normally play an important role in gut immunity, as their phenotype is controlled by the master transcription factor RORγt and their main effector cytokines are IL-17 and IL-22 [18]. On the other hand, differentiation of the functional phenotype Th2 is controlled by the master transcription factor GATA3, which is favoured by the action of IL-4, and their main effector cytokines are IL-13, IL-5 and IL-4. This effector phenotype not only plays a fundamental role in the orchestration of immunity against helminths and in allergy [19], but is also involved in the attenuation of neuroinflammatory processes and contributes to the consolidation of spatial memory under physiological conditions [20–24]. CD4^+^ T-cells can also acquire an anti-inflammatory functional phenotype, the T-regulatory phenotype (Treg), which can suppress the inflammatory function of effector T-cells [13]. Tregs cells are normally involved in the maintenance of tolerance toward self-constituents, limiting inflammatory responses against foreign antigens and, importantly, they are also known to be involved in the attenuation of neuroinflammation and consequent neurodegeneration [25–28]. During recent years, these functional phenotypes of CD4^+^ T-cells have been shown to participate in the physiopathology of neurodegenerative disorders. In this study, we focus on the analysis of the interplay between the different sub-populations of CD4^+^ T-cells and how they participate in regulating the outcome of neuroinflammation and neurodegeneration in the context of PD, AD, ALS and MS. Furthermore, in the last part of this review, we analyse the participation of peripheral monocytes/macrophages infiltrating the CNS, their interaction with T-cells and their contribution to the regulation of neuroinflammation.

### Role of T-cells infiltrating the CNS parenchyma in multiple sclerosis

**Figure 1:**
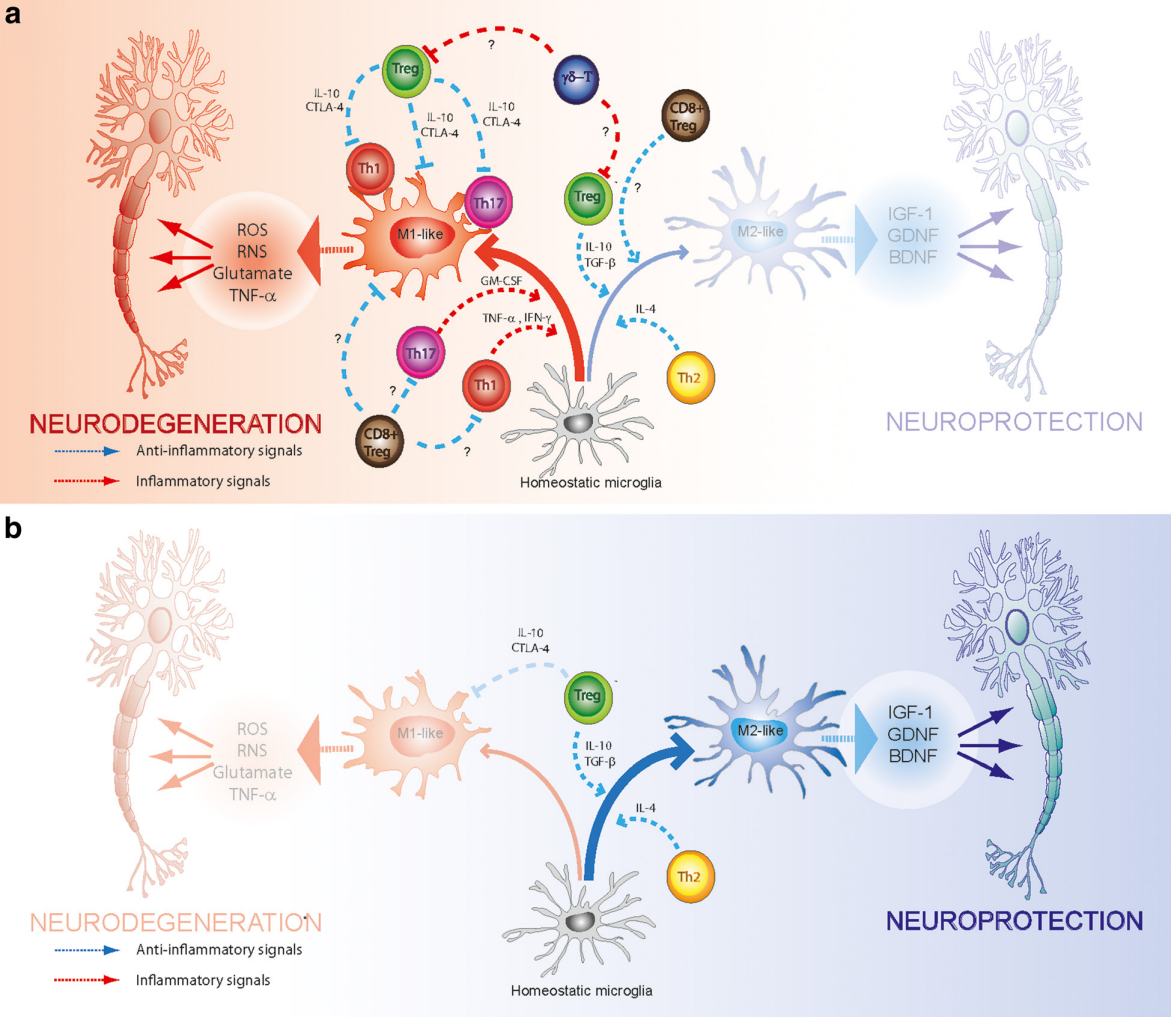
**T-cell-mediated regulation of neuroinflammation in neurodegenerative disorders.** The scheme shows how the participation of inflammatory and anti-inflammatory subsets of encephalitogenic T-cells might regulate microglial fate and, consequently, the degeneration or survival of neurons. Whereas Th1, Th17, GM-CSF-producer CD4^+^ T-cells and γδT-cells favour the acquisition of the neurotoxic M1-like phenotype by microglia (left side of the illustration), Th2, Tregs and some kinds of CD8^+^ T-cells can contribute to the promotion of neurosupportive M2-like phenotype in microglial cells (right side of the illustration). The scheme in **(a)** shows the 'natural' scenario during the progression of multiple sclerosis (MS), Parkinson's disease (PD) and Alzheimer's disease (AD), which involve the M1-like microglia and consequent neurodegeneration; however, upon disease remission or immunosuppressive interventions, it is possible to induce the M2-like phenotype of microglia and neuroprotection. In the case of PD and AD, only Th1, Th17 and Tregs cells have been associated with the regulation of neuroinflammation. The role of other T-cell subsets in the regulation of neuroinflammation associated to AD and PD remains unexplored. The scheme in **(b)** illustrates the participation of T-cells in the regulation of neuroinflammation associated to amyotrophic lateral sclerosis (ALS). The physiopathological scenario of this disorder 'naturally' involves the participation of encephalitogenic or meningeal Tregs and Th2 cells, which strongly contribute to the induction of the anti-inflammatory M2-like phenotype on microglial cells, thus slowing the progression of the neurodegenerative process. BDNF, brain-derived neurotrophic factor; GDNF, glial cell-derived neurotrophic factor; IGF-1, insulin-like growth factor 1; RNS, reactive nitrogen species; ROS, reactive oxygen species.

MS represent a neurodegenerative disease in which a T-cell-mediated response has been known to be involved for more than a decade. MS is a chronic demyelinating disease generated by an autoimmune response against constituents of the CNS. This autoimmune disease affects approximately 2.4 million individuals worldwide [29]. MS is characterised by the progressive loss of neurological function caused by the destruction of the axonal myelin sheath in several areas of the brain and the spinal cord, which is mediated, mainly, by self-reactive CD4^+^ T-cells [30]. The loss of myelin is manifested in clinical symptoms such as paralysis, muscle spasms, optic neuritis and neuropathic pain [31]. The pathological features of MS lesions involve BBB permeability, myelin sheath destruction, axonal damage, glial scar formation and the presence of inflammatory cells, mostly lymphocytes, infiltrated into the CNS [32]. The most used and accepted animal model equivalent of MS is experimental autoimmune encephalomyelitis (EAE), which corresponds to an induced autoimmunity in mice. EAE may be triggered in mice upon injection of peptides derived from myelin emulsified with adjuvant [33]. The administration of myelin-derived antigens in an immunogenic context induces the activation of self-reactive T-cells that are specific for myelin antigens, mediating myelin destruction. This induced autoimmunity is characterised by focal areas of demyelination along the brain and spinal cord, with axonal loss that results in ascending paralysis, affecting first the tail and then the hind limbs. Self-reactive CD4^+^ T-cells are activated by auto-antigen-presenting dendritic cells in peripheral lymph nodes and, then, the activated self-reactive CD4^+^ T-cells migrate into the CNS parenchyma. Self-reactive Th17 cells are the first T-cell population to infiltrate the CNS, as they express C-C chemokine receptor 6 (CCR6), which recognises C-C chemokine ligand 20 (CCL20) that is constitutively expressed on epithelial cells from the choroid plexus [34]. Th17 cells infiltrating the CNS are re-stimulated once inside the CNS by resident APCs, which is followed by microglia activation and production of IL-1β, TNF-α and IL-6, which contribute to myelin sheath damage [35]. This initial neuroinflammatory process results in BBB disruption and the entrance of leukocytes into the CNS parenchyma. Once T-cells enter the CNS, they are re-stimulated by resident APCs, such as astrocytes, microglia or infiltrated APCs such as dendritic cells and macrophages [36]. Thereby, APCs play an important role during the course of EAE, as they are involved in the peripheral activation of T-cells as well as in the re-stimulation of T-cells inside the CNS. Infiltration of T-cell into the CNS and their local re-stimulation lead to the manifestation of clinical symptoms [37]. Although there are several studies indicating that Th17 and Th1 cells infiltrate the CNS during the course of EAE, recent studies have shown that none of their signature cytokines are essential for the development of this disease. In contrast to the expression of IL-17 and IFN-γ, the production of granulocyte macrophage-colony-stimulating factor (GM-CSF) by autoreactive CD4^+^ T-cells has proven to be essential for the development of EAE [38,39]. The production of this cytokine is stimulated by IL-23 and by the expression of both transcription factors RORγt and Tbet, whereas it is inhibited by IL-12, IFN-γ and IL-27 [38,40]. On the other hand, Tregs progressively infiltrate the CNS, starting at disease onset. However, the generation of Tregs and the suppressive activity of CNS-infiltrated Tregs is impaired by γδT-cells [27]. γδT-cells infiltrate the CNS during EAE, starting at the onset of disease, reaching the highest cell number at the peak of disease manifestation before progressively disappearing from the CNS, thus correlating with the time course of EAE manifestation [27]. γδT-cells inhibit the function of Tregs cells and produce large amounts of IL-17 in response to IL-23. Once γδT-cells begin disappearing from the CNS, Tregs continue to progressively infiltrate the CNS and they can recover their suppressive activity, which correlates to the progressive attenuation of EAE manifestation [27]. Another kind of T-cell that is relevant for EAE is the CD8^+^ population. In this regard, some studies involving cell transfer have suggested the participation of CD8^+^ T-cell sub-populations in a pathogenic role of EAE [41]. On the other hand, there is strong evidence to indicate the participation of a regulatory sub-population of CD8^+^ T-cells, which play a beneficial role in EAE [42,43]. Studies show that EAE is more severe in mice that are deficient in or depleted of CD8^+^ T-cells, and the disease severity has been inversely correlated with the frequency of CD8^+^ T-cells infiltrating the CNS [41]. In this regard, distinct sub-populations of CD8^+^ Tregs have been described, including CD8^+^CD28^−^, CD8^+^CD122^+^ and CD8^+^LAP^+^ [44–46]. Importantly, the frequency of CD8^+^ T-cells is greater than that of CD4^+^ T-cells in inflamed plaques from MS patients, and CD8^+^ T-cells show oligoclonal expansion in plaque, cerebrospinal fluid and blood, suggesting an important role of this cell population in MS. Taken together, these data indicate the complex role of several T-cell populations in MS, which finally regulate microglial activation and, therefore, the outcome of neuroinflammation. An overview of the role of the different T-cell populations participating in neuroinflammation associated with MS is schematised in Figure 1.

### Involvement of T-cell-mediated immune response in Parkinson’s disease

PD is the second most common neurodegenerative disease after AD. PD is characterised by the loss of 50 to 70% of dopaminergic neurons located in the substantia nigra pars compacta (SN*pc*). The progressive degeneration of dopaminergic fibres in the brain results in prominent motor symptoms, such as bradykinesia, tremors, rigidity and postural instability. Although studied less in PD than in MS, the fundamental participation of T-cells has also been described in PD. An altered frequency of peripheral CD4^+^ T-cells has been described in blood samples obtained from PD patients [47,48]. Moreover, CD4^+^ and CD8^+^ T-cells that infiltrated into the brain parenchyma have been found in *post-mortem* samples obtained from PD patients [49,50], as well as in animal models using mice [50,51] and rats [52]. Recent studies carried out with T-cell receptor (TCR)-β-chain-deficient mice, SCID mice and recombination-activating-gen-1 (RAG1) knockout (RAG1KO) mice demonstrated that T-cell deficiency results in a strong attenuation of dopaminergic neurodegeneration in 1-methyl-4-phenyl-1,2,3,6-tetrahydropyridine (MPTP)-induced PD [50,53]. This reveals that T-cells are not only relevant, but are also required for neurodegeneration in PD. Additional experiments have shown that although CD8^+^ T-cell deficiency is negligible, the participation of CD4^+^ T-cells is fundamental for promoting the neurodegeneration of dopaminergic neurons in the SN*pc* of mice with PD [50]. These studies support the involvement of pathogenic CD4^+^ T-cell populations, which would induce the acquisition of an M1-like pro-inflammatory phenotype by the microglia, which is characterised by the secretion of inflammatory factors such as TNF-α, IL-1β, glutamate and superoxide [14,54]. Supporting the pivotal role of CD4^+^ T-cells potentiating microglial activation and favouring neurodegeneration in PD, it has recently been reported that a deficiency of class II major histocompatibility complex (MHC) results in attenuation of both microgliosis and loss of dopaminergic neurons in a mouse model of PD [55]. Experiments addressing the phenotype of pathogenic CD4^+^ T-cells involved in PD have shown that both Th1 and Th17 autoreactive cells are important for the promotion of neuronal loss [26]. Addressing the molecular mechanisms involved in CD4^+^ T-cell-mediated loss of dopaminergic neurons in PD, a study has shown that the participation of Fas-FasL interactions seems to contribute to the neurodegenerative process [50]. Importantly, we have recently demonstrated that dopamine receptor D3 (D3R), expressed in CD4^+^ T-cells, is fundamental in inducing the loss of dopaminergic neurons in the SN*pc* of a PD mouse model [17]. In this regard, we and others have reported that D3R-deficient (D3RKO) mice are resistant to MPTP-induced PD [17,56]. Interestingly, when wild type (WT) CD4^+^ T-cells were transferred to D3RKO mice, the animals acquired the capability to respond to MPTP-induced neurodegeneration. On the other hand, RAG1KO mice, which are devoid of T-cells and are resistant to MPTP-induced PD, acquire the capability to respond to MPTP-induced neurodegeneration when WT CD4^+^ T-cells were transferred, but not when D3RKO CD4^+^ T-cells were transferred [17]. Notably, our data indicate that the stimulation of D3R expressed on CD4^+^ T-cells favours the acquisition of Th1 inflammatory cells, thus indicating the crucial importance of this pathogenic phenotype in the CD4^+^ T-cells immune response that is involved in PD [17]. In this regard, we observed that WT, but not D3RKO, CD4^+^ T-cells infiltrating the SN*pc* during MPTP-induced PD produced high levels of IFN-γ and TNF-α, which are two cytokines that act synergistically in microglia, promoting the inflammatory M1-like phenotype [57]. Thus, these findings point towards the important role of CNS-derived dopamine in the regulation of T-cell-mediated immunity during neuroinflammation. Conversely, other T-cell subsets, such as Tregs and Th2, could contribute to microglial acquisition of an M2-like anti-inflammatory phenotype, which release neurotrophic factors, including IGF-1, promoting neuronal protection [14,26]. Indeed, it has been demonstrated that Tregs elicit neuroprotection for dopaminergic neurons of SN*pc* in MPTP-induced PD in mice [26,58]. Further analyses have shown that these Tregs cells act directly on activated M1-like microglial cells, attenuating migration, phagocytosis and the production of neurotoxic factors [58,59]. *In vitro* experiments have shown that Tregs-mediated inhibition of M1-like microglia functions occurred through the suppression of NF-κB activation and required not only soluble mediators from Tregs, but also cell-cell contacts with microglial cells [59]. Another group of studies have also shown evidence of the neuroprotective role of Tregs in PD. In these studies, copolymer-1 was used as an immunogen, which is a potent inducer of encephalitogenic Tregs [60]. It has been shown that adoptive transference of CD4^+^ T-cells isolated from mice, immunised with copolymer-1, attenuates the neurodegeneration of dopaminergic neurons in SN*pc* of mice with MPTP-induced PD [61]. On the other hand, Reynolds *et al*. found that Th2 cells, which are specific against CNS antigens involved in PD, do not play a relevant role, exacerbating or attenuating neurodegeneration in MPTP-induced PD [26]. Other encephalitogenic T-cell subsets described as playing important inflammatory or anti-inflammatory roles in MS have not yet been studied in PD. Thus, future efforts are necessary to elucidate the participation of anti-inflammatory CD8^+^ T-cell subsets, inflammatory γδT-cells and GM-CSF-producer T-helper cells in the physiopathology of PD. An integrative summary of the role of T-cells in the neurodegenerative process associated with PD is shown in Figure 1.

### T-cell-mediated immune response associated with Alzheimer’s disease

AD is the most common neurodegenerative disorder worldwide, and it leads to irreversible cognitive impairment and important behavioural alterations. Similar to PD, T-cells have been detected as infiltrating the brain parenchyma in close proximity with Aβ deposits in *post-mortem* samples of AD patients and in mouse models of AD [62,63]. Consistent with this observation, Aβ-reactive T-cells have been found in peripheral blood obtained from AD patients [64]. Interestingly, Aβ-specific T-cell response was highly dependent on a particular epitope. IFN-γ has been shown to play an important role in T-cell-mediated responses involved in AD. Limited expression of IFN-γ in the brain favoured T-cell infiltration into the CNS parenchyma and the formation of immunosynapses with microglia in a mouse model of AD [62]. Moreover, recent data indicate that IFN-γ produced by T-cells infiltrating the brain in AD favour increased microglial activation, Aβ deposition and impaired cognitive functions [16]. Indeed, systemic administration of anti-IFN-γ antibody in mice with AD decreased microglial activation and Aβ deposition, and improve cognitive functions [16]. Interestingly, vaccination with Aβ peptide has proved efficacious in AD mouse models [65]. In this regard, transcutaneous immunisation with aggregated Aβ plus the adjuvant cholera toxin results in a high titre of anti-Aβ antibodies and a significant decrease in cerebral Aβ aggregated in mice with AD [24]. The fact that most anti-Aβ antibodies were of IgG1 isotype in this vaccine can be explained by the induction of a Th2-mediated immune response using this therapeutic approach; this represents a kind of immune response that promotes a switch-on microglial M2-like phenotype with consequent neuroprotection [14,21,25,66,67]. Notably, in the study carried out by Nikolic *et al*., they did not detect T-cell infiltration in the brain parenchyma [24]. Thereby, it is possible that, when Th2 responses are induced against CNS antigens, CD4^+^ T-cells mainly act from meningeal space, affecting glial function without infiltrating the brain parenchyma, as seen in healthy animals [21,66]. The role of Tregs has also been addressed in AD. The adoptive transference of Tregs significantly ameliorates impaired cognition and reduces the Aβ-deposits and microglial activation in a mouse model of AD [25]. Moreover, higher Tregs function has been associated with lower cognitive manifestation in AD patients [68]. Addressing the participation of different inflammatory phenotypes of CD4^+^ T-cells infiltrating the brain during AD, studies indicate an important role for both Th1 and Th17 cells. In this regard, Browne *et al*. examined the role of Aβ-specific CD4^+^ T-cells on Aβ accumulation in transgenic mice that overexpress amyloid precursor protein and presenilin-1. The results revealed that Aβ-specific Th1 and Th17 cells infiltrate the brain in this AD model. To address the relevance of the different CD4^+^ T-cell phenotypes in AD, the same authors generated Aβ-specific CD4^+^ T-cells by immunisation of WT mice with Aβ and a TLR agonist, and then polarised them *in vitro* towards Th1, Th2 or Th17, which were then adoptively transferred into mice with AD. Their results showed that Th1 cells, but not Th2 or Th17 cells, increased microglial activation and Aβ deposition, and these changes were associated with impaired cognitive function [16]. Thus, this data indicates a leading role of inflammatory Th1 cells in the immune response associated with AD. However, there is a group of studies supporting the relevance of the participation of Th17 cells in the immune response associated with AD. In this regard, the phenotypic analysis of circulating T-cells obtained from AD patients has shown higher frequencies of the activation marker CD25 and of CCR6, which is characteristic of Th17 cells [69], an inflammatory phenotype associated with neuronal damage in MS and PD [26,30]. Moreover, significant infiltration of Th17 cells has been detected in the hippocampus by the immunodetection of the master transcription factor RORγt and the expression of Th17 cytokines IL-17 and IL-22, which have been associated with neuron loss and gliosis in cornu ammonis area 1 (CA1) in a rat model of AD [70]. Furthermore, immunohistochemical analyses in brain sections of AD animals showed a high expression of Fas in the hippocampal neurons and a high expression of FasL in Th17 cells that were in close proximity with neurons. Thus, these findings suggest that Th17 cells, which were infiltrated into the hippocampus parenchyma, participate in neuroinflammation and neurodegeneration of AD by releasing pro-inflammatory cytokines and by inducing neuron apoptosis mediated by Fas-FasL contacts [70]. Taken together, the current evidence indicates that inflammatory Th1 and Th17 CD4^+^ T-cells play an important role in the physiopathology of AD, favouring the disease progression (Figure 1). Nevertheless, other important encephalitogenic T-cell subsets that play important inflammatory or anti-inflammatory roles in MS have not yet been studied in AD. Furthermore, studies implementing the genetic deletion of specific subsets of inflammatory or anti-inflammatory T-cells would be very helpful in order to clarify the specific roles and relevance of these different T-cell subsets in the development and progression of AD. Therefore, similar to the case for PD, future efforts are required to evaluate the participation of important T-cell subsets, such as γδT-cells and GM-CSF-producer T-helper cells, in the physiopathology of AD.

### Involvement of T-cell function in the pathogenesis of amyotrophic lateral sclerosis

ALS is a neurodegenerative disorder characterised by selective and progressive degeneration of upper and lower motor neurons, leading to dramatic muscle paralysis and death. The initial cause of ALS remains unclear, but nearly 2% of patients have mutations in the *Cu/Zn SOD1* gene, which codes for a ubiquitous protein that acts in removing dangerous superoxide radicals from inside the cell. It has recently been proposed that both loss of dismutase activity and protein aggregation of SOD1 are involved in triggering neuronal loss in ALS [12]. Transgenic mice that overexpress human mutant SOD1 (mSOD1) develop motor pathology resembling ALS [71]. Similar to that occurring in other neurodegenerative diseases such as PD, AD and MS, neuroinflammation is a prominent feature in the process of neuronal loss in ALS patients, as well as in animal models of ALS [1]. Accumulating evidence has emerged during recent years, indicating that the adaptive immune system plays an important role in regulating the progression of ALS [20,23,72]. Interestingly, T-cell infiltration in spinal cords of ALS mice has been described in close proximity with dying motor neurons and has been associated with glial activation [20,23,72]. Nevertheless, the exact role of the direct interaction of T-cells with motor neurons in ALS remains unclear. Studies addressing the role of T-cells in ALS have shown that T-cell-deficient mice with ALS, generated by crossing mSOD1 transgenic mice with RAG2KO or with TCR β-chain knockout (TCRβKO) mice, develop an accelerated progression to the symptomatic stage of ALS [20,23]. Furthermore, T-cell deficiency in mice models of ALS is associated with accelerated neuronal loss and decreased glial activation. Moreover, the reconstitution of mSOD1/RAG2KO mice with functional T-cells has shown to recover a prolonged survival of ALS mice, increased glial activation and delayed neuronal loss [20]. Further experiments have shown that mSOD1/CD4KO mice display a phenotype resembling that observed in mSOD1/RAG2KO mice, thus attributing the neuroprotective role to CD4^+^ T-cells, but not to CD8^+^ T-cells or B-cells [20]. Importantly, ALS mice show microgliosis and astrogliosis in the spinal cord, and T-cell deficiency does not significantly affect astrogliosis. However, when lacking T-cells or, specifically, CD4^+^ T-cells, ALS mice display microglial cells with attenuated morphological microgliosis, decreased activation markers such as CD11b and CD68, increased levels of pro-inflammatory cytokines such as IL-6 and TNF-α, decreased levels of trophic factors such as IGF-1, GDNF and BDNF as well as elevated levels of nicotinamide adenine dinucleotide phosphate (NADPH) oxidase 2, which is known to enhance microglial release of ROS [20,23]. All these data indicate that CD4^+^ T-cells play a regulatory role on microglial cells during ALS progression, providing supportive neuroprotection by favouring the acquisition of the M2-like phenotype by microglial cells. Recent evidence suggests that both Tregs as well as Th2 cells actively contribute to the neuroprotective effect exerted by CD4^+^ T-cells over microglia. In this regard, analysis of peripheral blood CD4^+^ T-cells obtained from ALS patients has shown decreased levels of Tregs and a decreased level of protein FoxP3 expression, both of which result in a lower suppressive activity [73]. Both parameters were reduced in rapidly progressing ALS patients, and inversely correlated with the progression rates [72,74,75]. In addition, both the Th2 cytokine IL-4 and the master transcription factor controlling Th2 phenotype, GATA3, also became reduced in rapidly progressing patients, inversely correlating with the progression of motor symptoms [74]. Furthermore, supporting the participation of Th2 cells during ALS progression, elevated IL-4 levels were found in spinal cords that were obtained from mSOD1 mice [23]. Moreover, stimulation of microglial cells from healthy or ALS mice with IL-4 induces a strong expression of IGF-1 and attenuates the production of inflammatory cytokine IL-6 [23]. Thus, current evidence collectively suggests that, by regulating the acquisition of the functional phenotype of microglia, Tregs as well as Th2 lymphocytes would play an important neuroprotective role during ALS progression. See Figure 1 for an integrative overview of the role of T-cells in neuroinflammation in ALS.

### Contribution of macrophages to the neuroinflammatory process associated with neurodegenerative diseases

Current evidence indicates that microglial cells play a key role in sensing protein aggregates in a TLR-dependent manner in the CNS of patients suffering neurodegenerative diseases [6,7]. This initial activation of microglia produces pro-inflammatory mediators, favouring BBB permeabilisation and, therefore, the infiltration of peripheral leukocytes into the CNS, including T-cells, macrophages and others [76]. Macrophages share several functional features with microglia, including: i) the expression of TLRs and, therefore, the capability of being activated by aggregated proteins or pathogen-associated molecular patterns [6,7], ii) the expression of class II MHC and, therefore, the capability to present antigens to CD4^+^ T-cells and exert an influence on the functional phenotype of T-cells [77], and iii) the ability to polarise their functional phenotype towards an inflammatory M1 phenotype versus an anti-inflammatory M2 phenotype, which can be influenced by inflammatory T-cells and Tregs [78]. Thus, when the BBB is already permeabilised, the possibility arises in which peripheral macrophages may acquire a relevant role in the outcome of neuroinflammation. In fact, there is also evidence suggesting that a strong inflammatory response in the periphery, such as systemic lipopolysaccharide (LPS) [79] or viral infections [80], may result in subsequent infiltration of peripheral leukocytes into the CNS with consequent neuroinflammation and neurodegeneration. Therefore, irrespective of where the inflammatory response is initiated (periphery versus CNS), macrophages infiltrating the CNS can acquire a relevant role in the outcome of neuroinflammation associated with neurodegenerative disorders. According to this notion, there are a number of studies associating neurodegenerative disorders with alterations in molecular components, determining the M1/M2 behaviour of peripheral macrophages. In this regard, the anti-inflammatory surface molecule CD200R is decreased in monocyte-derived macrophages obtained from the peripheral blood of PD patients in comparison with those from healthy donors [81]. Interestingly, the same authors found that inducible expression of CD200R in monocyte-derived macrophages correlated inversely with the onset age of PD and with the capability of these cells to produce TNF-α [81]. According to the important role of chemoattraction of monocytes in inflamed tissues, as well as their relevance in neurodegenerative disorders, the polymorphism of monocyte chemoattractant protein 1 (or CCL2) has been associated with the age of PD patients at onset [82]. Similarly, a study found that peripheral blood monocytes obtained from PD patients displayed a strong up-regulation of the surface CCR2, which is the receptor for the chemokine CCL2 [83]. Furthermore, according to the relevance of monocytes/macrophages in PD, a recent study has shown that the transference of GDNF-transfected macrophages into mice undergoing 6-hydroxydopamine-induced PD results in a potent neuroprotective effect [84]. Another study addressing the analysis of these alterations in gene-expression profiling of peripheral blood mononuclear cells obtained from patients undergoing sporadic ALS found an up-regulation of TLR4-signaling-associated genes [85], thus suggesting a chronic activation of peripheral blood monocytes/macrophages in ALS. According to this idea, Butovsky *et al*. have found that monocytes obtained from an ALS mouse model present a polarised M1 phenotype and increased CCR2 expression [86]. The same authors have shown that the treatment of ALS mice with anti-Ly6C antibodies results in a partial depletion of monocytes, decreased infiltration of M1 macrophages in the spinal cord and attenuated neurodegeneration [86], thus indicating a pivotal role of monocytes/macrophages in the physiopathology of ALS. Similar to the inflammatory phenotype observed in monocytes/macrophages from PD and ALS patients, studies addressing the characterisation of peripheral blood monocytes/macrophages of AD patients have shown that these cells display an increased M1 phenotype, with increased expression of chemokine receptors CCR2 and CXCR1 [87,88], and stronger production of inflammatory cytokines, including IL-1β, IL-6 and IL-23 [88,89]. Regarding MS, studies analysing the M1/M2 profile of monocytes/macrophages obtained from human blood samples are more difficult to interpret, as many studies are performed with relapsing-remitting patients and/or individuals treated with the immunomodulatory cytokine IFN-β. However, the relevance of the M1/M2 profile of monocytes/macrophages has been addressed in EAE. In this regard, M1 macrophages have been found in the early stage of acute EAE, whereas the frequency of M2 macrophages is increased during the peak of disease manifestation and throughout the recovery stage [90]. Indeed, a reduced M1/M2 ratio in the profile of monocytes/macrophages in the blood, as well as in the CNS, promotes an attenuated manifestation of the disease, whereas an increased M1/M2 ratio favours relapsing EAE [91]. Furthermore, the administration of *ex vivo*-activated M2 monocytes both reduces the severity of ongoing EAE and favours the expression of immunosuppressive molecular profile in CNS lesions [91]. In addition, experiments carried out in an animal model of focal demyelinisation have shown that M2 macrophages promote remyelinisation by inducing oligodendrocyte differentiation through a mechanism involving the secretion of activin-A [92]. Importantly, a study has shown that GM-CSF production by encephalitogenic CD4^+^ T-cells is absolutely necessary for EAE manifestation, and its cellular targets were myeloid cells expressing the GM-CSF receptor [38]. Furthermore, experiments performed with bone-marrow chimeras, generating animals with selective deletion of the GM-CSF receptor in microglia or in CNS-infiltrating myeloid cells, have demonstrated that the GM-CSF receptor was required to be expressed in myeloid cells from a peripheral origin, but not in microglia, in order to allow EAE manifestation [38]. Taken together, all of these data indicate that peripheral monocytes/macrophages display an inflammatory M1 phenotype in human individuals suffering neurodegenerative disorders. Moreover, evidence obtained from animal models suggests that these cells may be relevant in neuroinflammation and in the progression of neurodegeneration, and their manipulation favouring the M2 phenotype has shown promising therapeutic potential.

## Conclusions

Neuroinflammation constitutes a critical process in the physiopathology of several neurodegenerative disorders, which is required for the progression of neurodegeneration. Differential activation of microglial cells constitutes a central point of regulation in neuroinflammation, which can result in neurotoxic or neuroprotective environments that are critical for the fate of neurons. Growing evidence has indicated a fundamental role of CD4^+^ T-cells in the regulation of neuroinflammation and consequent neurodegeneration in a number of neurodegenerative diseases. In this regard, encephalitogenic inflammatory CD4^+^ T-cells such as Th1, Th17, GM-CSF-producer CD4^+^ T-cells and γδT-cells strongly contribute to feed chronic neuroinflammation, thus perpetuating neurodegenerative processes. In contrast, encephalitogenic or meningeal Tregs and Th2 cells decrease inflammatory functions in microglial cells and promote a neurosupportive microenvironment. Interestingly, whereas some neurodegenerative disorders such as MS, PD and AD involve the participation of inflammatory CD4^+^ T-cells 'naturally', the physiopathology of other neurodegenerative diseases, such as ALS, is associated with the participation of anti-inflammatory CD4^+^ T-cells that delay the neurodegenerative process. Thus, current evidence supports the hypothesis that the involvement of a CD4^+^ T-cell-mediated response against CNS antigens constitutes a key component for the progression of the neurodegenerative process. Furthermore, an increasing number of studies have shown the relevance of peripheral monocytes/macrophages in the progression of neuroinflammation and their therapeutic potential for neurodegenerative disorders. According to this notion, immunoregulatory strategies to attenuate inflammatory CD4^+^ T-cells or M1 monocytes/macrophages and to strengthen the function of M2 monocytes/macrophages or Tregs specific for CNS antigens involved in neurodegenerative disorders should be considered in future therapies.

## Abbreviations

Aβ: β-amyloid peptide
AD: Alzheimer’s disease
ALS: amyotrophic lateral sclerosis
APCs: antigen-presenting cells
BBB: blood-brainbarrier
BDNF: brain-derived neurotrophic factor
CA1: cornu ammonis area 1
CCL*n*: C-C chemokine ligand *n*
CCR*n*: C-C chemokine receptor *n*
CD*n*: cluster of differentiation *n*
CD4KO: CD4 knockout
CNS: central nervous system
D*n*R: dopamine receptor *n*
D*n*RKO: D*n*R knockout
EAE: experimental autoimmune encephalomyelitis
GDNF: glial cell-derived neurotrophic factor
GM-CSF: granulocyte macrophage-colony stimulating factor
IFN-γ: interferon-γ
IGF-1: insulin-like growth factor 1
IL-*n*: interleukin-*n*
LPS: lipopolysaccharide
MHC: major histocompatibility complex
MPTP: 1-methyl-4-phenyl-1,2,3,6-tetrahydropyridine
MS: multiple sclerosis
mSOD1: mutant SOD1
NADPH: nicotinamide adenine dinucleotide phosphate
NF-κB: nuclear factor kappa-light-chain-enhancer of activated B-cells
PD: Parkinson’s disease
RAG*n*: recombination-activating-gen-*n*
RAG*n*KO: RAG*n* knockout
RNS: reactive nitrogen species
ROS: reactive oxygen species
SN*pc*: substantia nigra pars compacta
SOD1: superoxide dismutase 1
TCR: T-cell receptor
TCRβKO: TCR β-chain knockout
Th*n*: T-helper *n*
TLR: toll-like receptor
TNF-α: tumor necrosis factor-α
WT: wild type
